# Load Controlled Fatigue Behaviour of Microplasma Arc Welded Thin Titanium Grade 5 (6Al-4V) Sheets

**DOI:** 10.3390/ma13225128

**Published:** 2020-11-13

**Authors:** Jaroslaw Szusta, Nail Tüzün, Özler Karakaş

**Affiliations:** 1 Faculty of Mechanical Engineering, Bialystok University of Technology, 45C Wiejska Str., 15-351 Bialystok, Poland; j.szusta@pb.edu.pl; 2 Department of Mechanical Engineering, Faculty of Engineering, Pamukkale University, Kinikli, 20160 Denizli, Turkey; ntuzun12@posta.pau.edu.tr

**Keywords:** titanium alloy, microplasma arc welding, fatigue, load controlled

## Abstract

The current study investigates the load controlled fatigue properties of the microplasma arc welded thin titanium Grade 5 (6Al-4V) sheets. In order to explore the effect of weld geometry on the fatigue, two different welded joints were used in the experimental studies. Load controlled fatigue test results were evaluated to present an outlook on the behaviour of microplasma welded titanium alloy Grade 5 sheets under cyclic loading. Even though the previously published monotonic tests showed successful use of microplasma arc welding to join thin titanium Grade 5 sheets with mechanical properties comparable to the base metal, fatigue life of the welded joints was lower than the lives of samples without welds. In particular, the fatigue performance of overlap joints was very poor. This was presumed to be due to the changed material properties of the heat affected zone which was formed by the excess heat of the welding process as fractures often occurred at such locations. Based on experimental findings and fractographic observations, a clear adverse effect of welding process in material behaviour was discovered. Despite the concentrated heat of microplasma arc welding, post-weld heat treatment of the weld area is recommended to improve the mechanical behaviour of the welded joints.

## 1. Introduction

As is commonly known, titanium alloys are often preferred in industry for their relatively high specific strength and corrosion resistance, especially in industries where the weight of the components is very important such as aerospace and marine engineering constructions [1,2,3]. In addition, due to the biocompatibility of titanium, it is often used in the making of medical implants [4].

In continuation to the authors’ previous study [5], which investigated the monotonic material properties of welded thin titanium alloy Grade 5 (6Al-4V) samples, the same material was selected once again because of its availability and wide range of applications. In order to manufacture the welded test samples from the chosen material, microplasma arc welding was used. As the name suggests, this welding method is a low current variation of widely used plasma arc welding with a specialized, highly precise plasma torch. The advantage of microplasma arc welding method is a very concentrated plasma arc and welding heat. Therefore, thin sheets which are relatively more susceptible to heat-related welding distortions and adverse effects of the heat-affected zone (HAZ) can be welded with microplasma arc welding.

There is considerable amount of fatigue studies regarding titanium alloys. However, studies that focus on the fatigue of microplasma arc welding are quite scarce. In order to provide an outline of the state of the art, articles that are related to the current study are presented briefly.

The influence of microstructure on the fatigue of welded titanium Grade 5 specimens were examined with load controlled fatigue tests and damage initiation, damage propagation and fracture behaviour were observed in [6]. In [7], service lives of gas tungsten arc, electron beam and laser beam welded titanium Grade 5 were investigated by evaluating the fatigue crack growth parameters of samples. Detailed fatigue test results of titanium Grade 2 and Grade 5 specimens joined with laser and hybrid welding were presented in [8]. Crack propagation of titanium Grade 5 specimens manufactured using Selective Laser Melting under constant amplitude loading was investigated in [9]. Notched titanium Grade 2 samples were subjected to uniaxial-tension load controlled fatigue tests at 500 °C and results were reported in [10]. Bimodal harmonic structured Grade 5 titanium was investigated under four-point bending in [11] and the effects of the material microstructure on small fatigue crack propagation were observed. In [12], diffusion bonding was used to manufacture porous pure titanium samples with 50–70% porosity, and experimental studies were performed to observe out-of-plane compression fatigue behaviour of the samples. In [13], the fatigue behaviour of Ti-6Al-4V ELI specimens was studied with and without mean strain/stress effect using four different strain ratios. Commercial purity titanium tubes were welded using fibre laser welding and semiautomatic GTAW welding, and microstructural and fatigue resistance comparisons between different welding methods were presented in [14]. Changes in microstructure of laser-welded titanium Grade 5 plates were observed in [15] and the effects of microstructure on fatigue crack growth rates were investigated. In [16], laser powder bed and electron beam melted titanium Grade 5 were investigated in terms of monotonic and cyclic mechanical properties while taking into account different surface roughness and heat treatments. In [17,18], detailed monotonic and cyclic investigations on butt welded AA6056 and Ti6Al4V using laser beam welding were performed. Titanium Grade 2 butt-welded joints, transverse fillet welded joints and longitudinal fillet welded joints were subjected to fatigue tests in [19], in order to improve the use of titanium in marine engineering. Additionally, it is worth mentioning fatigue studies presented in [20,21,22,23,24,25,26] for serving as an inspiration and example for the current study even though different materials were studied.

In the present study, the fatigue of titanium Grade 5 samples with microplasma arc welded joints was investigated by evaluating the behaviour of the material under load controlled cyclic tests. The aim of the study was to achieve a basic level of understanding regarding fatigue of titanium welded joints. While there are some studies regarding the fatigue behaviour of titanium, as cited previously, the present study investigated the microplasma arc welding as a potential means of overcoming some of the more common adverse effects of the welding process (e.g., HAZ with poor fatigue behaviour). For this investigation, one sample without weld and two types of welded samples made of 1 mm thick titanium Grade 5 sheets were subjected to load controlled fatigue tests. The specimen types were sample without weld (Type 1), butt-welded sample (Type 2), and overlap welded sample (Type 3) [5]. Fatigue parameters of each type of sample were determined based on experimental results. In addition, metallographic and fractographic examinations were presented. The findings of the tests were presented along with relevant evaluations.

## 2. Materials and Methods

As it was disclosed previously, details about the material and samples used in this research were previously published in detail in [5]. However, brief information regarding the material and test samples are presented in their respective subsections for the sake of completeness.

### 2.1. Material

**Figure 1:**
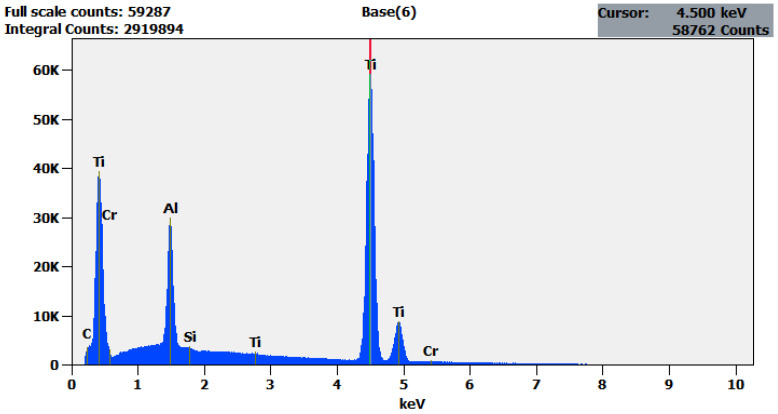
EDS analysis of Titanium Grade 5.

Analysed chemical composition of sheet material (titanium Grade 5) used in the experiments is given in Table 1. A HITACHI S-3000N scanning electron microscope (SEM) (Tokyo, Japan) with the X-ray microanalysis adapter—EDS type QUEST from THERMO NORAN (Waltham, MA, USA)—was used to determine the chemical composition of the material. EDS analysis spectra are also provided in Figure 1. The measurements were carried out in a plane parallel to the length of the sample in the middle of it along its thickness.

### 2.2. Research Samples

**Figure 2:**
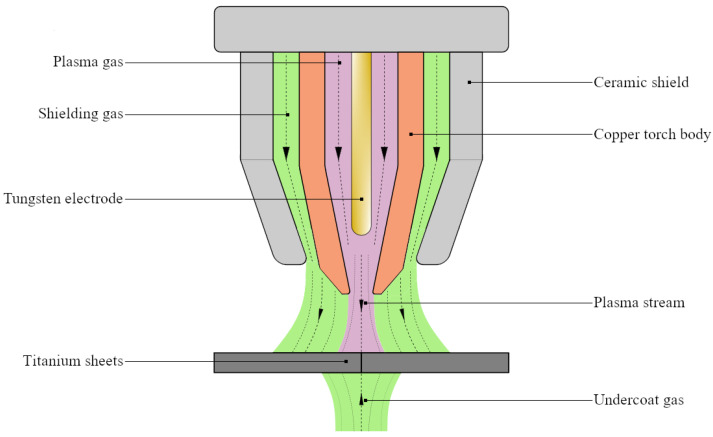
Simplified schematic of microplasma arc welding.

In order to obtain dimensional consistency between the samples and test results, titanium specimens were manufactured following a set of procedures. First, cutting off the initial shapes of the elements were carried out using the laser plotter from the rolled titanium sheets with their longitudinal axis coinciding with the rolling direction. Next, the sample elements were secured in position in the welding machine before they were joined by welding of their edges using microplasma arc welding under protective gas (helium) without the use of filler material. The welding machine used in the process was automated with a set welding speed. Figure 2 presents a simplified schematic of microplasma arc welding process. Cut plates were aligned on the welding device and fixed in position during welding with the use of bolts and a support device. Alongside the automated welding process, this prevented potential misalignments that might occur during welding. The welding parameters used for joining sample elements are listed in Table 2.

**Figure 3:**
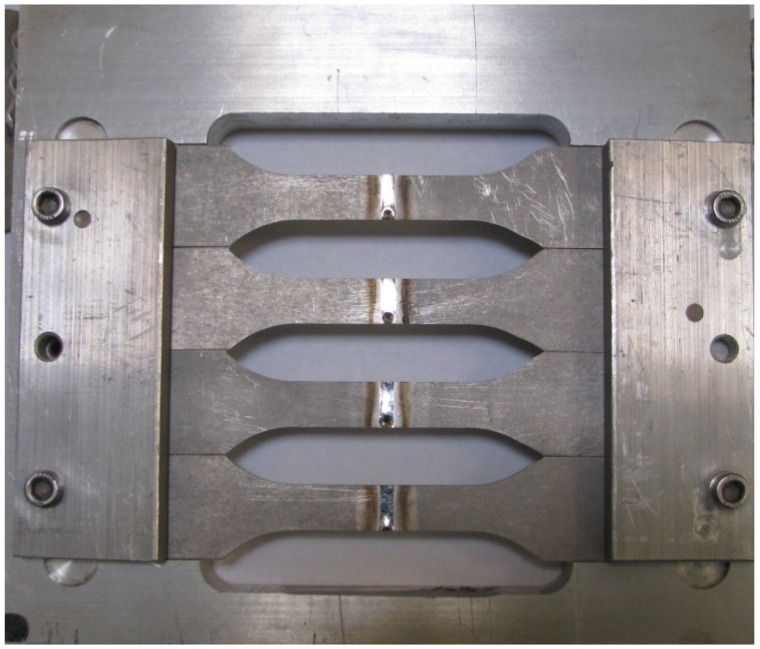
Butt welded test samples (type 2) fixed positioning device.

Finally, the welded samples were positioned using a device specially designed to align the samples and cut in the standard “paddle” shape using a waterjet cutting machine. The cut samples fixed in the positioning device are presented in Figure 3. Type 2 samples were samples with simple butt welds to joint opposing plates. Type 3, however, were samples consisting of two plates overlapping each other for 30 mm with fillet welds jointing each end of the plate on both sides.

### 2.3. Monotonic Tensile Tests

**Figure 4:**
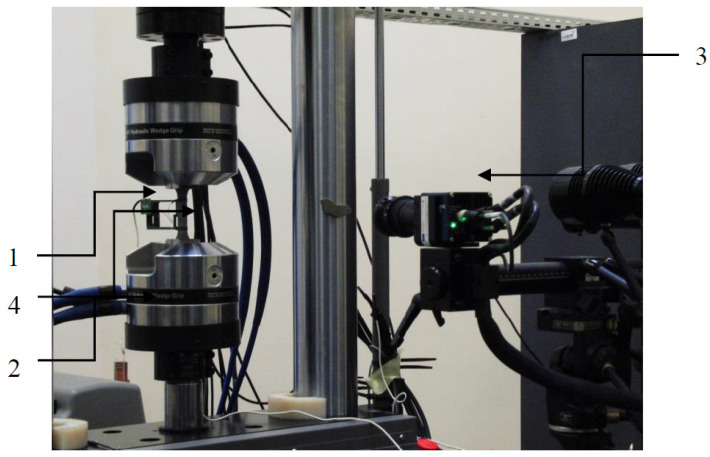
Experimental setup and the sample [5].

Monotonic tests were carried out according to the standard of PN-EN ISO 6892-1:2016-09 [27] and with a constant speed of Δl=0.001 mm/s and. The tests were controlled by using an Epsilon 50 linear extensometer with a 50 mm base (1) and monotonic loading was applied with a servo-hydraulic strength machine MTS 809 A/T (Eden Prairie, MN, USA) (2). In addition to the extensometer, sample deformation was measured with the ARAMIS 4M vision system (3). Experimental setup, along with the test sample (4) is shown in Figure 4. Throughout the test, the mechanical properties of the samples were monitored and recorded. Furthermore, GOM ARAMIS 4M vision system (Braunschweig, Germany) was used to observe sample deformation via the digital image correlation (DIC) method. Each sample type was tested three times and an arithmetic mean of 3 repeats was used to determine the monotonic properties of each, respectively.

### 2.4. Load Controlled Fatigue Tests

The same experimental setup that was utilized for monotonic tensile tests was also used for fatigue tests with the exception of the ARAMIS 4M vision system. Due to the unpredictable nature of fatigue tests and the file size restrictions of the computer system used for recording, it was not plausible to use the vision system during fatigue tests effectively. Test samples of titanium welded joints (Type 2 and Type 3) have been subjected to load controlled fatigue tests. Since Type 3 samples were welded with an overlap joint, clamping the sample to the test setup directly would cause bending. In order to prevent this, small pieces of titanium of same 1 mm thickness was glued using epoxy resin to each end of the sample. With this, it was possible to clamp the sample parallel to the grips normally, as if it is a 2 mm tensile sample. Levels of given loads were determined on the basis of the maximum values of stresses acquired from the monotonic tests of samples. The samples were force loaded with a unidirectional stress of $R_{\sigma }=0$. The following load levels were adopted: $0.92\;\sigma_{\mathrm{UTS}}$; $0.82\;\sigma_{\mathrm{UTS}}$; $0.72\;\sigma_{\mathrm{UTS}}$; $0.5\;\sigma_{\mathrm{UTS}}$. The test was performed three times for each of the load levels and sample type and the values were calculated as the arithmetic mean of the obtained results.

## 3. Results and Discussions

Based on the monotonic tensile test results, the following material parameters were determined from the monotonic tests; Young’s modulus E, yield strength *R*_YP_, strength limit *R*_UTS_, stress at fracture *R*_U_, elongation *A*, necking *Z*. These monotonic material parameters were presented in Table 3.

Moreover, the Vickers method was used to measure the hardness of titanium welded joints in multiple locations of the specimens. Sinowon Vickers Hardness Tester HV-50A (DongGuan, China) with a 5 kg indenter load was used to perform the hardness tests. The measurements were carried out on the unaffected base metal (UBM), in the heat-affected zone (HAZ) and in the centre of the weld (W). Hardness measurements were carried out for both welded sample types. Table 4 presents the results of the hardness tests which are the arithmetic mean of the three different measurements from each location.

**Figure 5:**
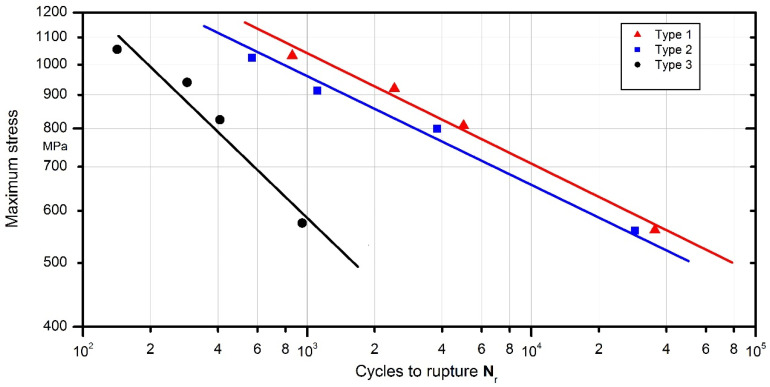
Wöhler lines of load controlled fatigue tests of Type 1–3 samples.

As mentioned previously, the samples (Type 1, Type 2 and Type 3) were subjected to force controlled cyclic loading with a stress ratio of $R_{\sigma }=0$. The following load levels were adopted: $0.92\;\sigma_{\mathrm{UTS}}$; $0.82\;\sigma_{\mathrm{UTS}}$; $0.72\;\sigma_{\mathrm{UTS}}$; $0.5\;\sigma_{\mathrm{UTS}}$. Table 5 presents the values of the set load levels and the measurement results obtained during the test. Based on these results, Wöhler lines of load controlled fatigue tests are plotted and presented in Figure 5.

When results of each sample type are compared, the lower fatigue life of specimens is evident. The gap between the fatigue lives of each specimen becomes larger at lower load levels, with Type 1 specimens having the greatest number of cycles to failure. Type 3 specimens had the lowest number of cycles to failure with a significant disparity compared to the fatigue lives of other specimens. When Wöhler line of each specimen is compared, the very steep slope of the line corresponding to Type 3 samples can be observed. Fatigue strength of Type 3 samples rapidly decreases with each number of cycles. In contrast, although Type 2 samples still had slightly lower fatigue life compared to Type 1, the slope of its Wöhler line was comparable to Type 1. The weld behaviour that occurs due to change in material and geometry can be treated as a “notch”. This supposed notch is not necessarily physically observable but rather inferred from the behaviour of the sample. It can be said that the steeper slopes of Wöhler lines correspond to a sharper notch. However, it should be noted that slopes alone are not enough to quantify this supposed sharpness of the notch and it requires further numerical investigation in order to determine accurately, which was considered the topic of future investigations using fatigue evaluation methods and was not performed as part of the current study.

**Figure 6:**
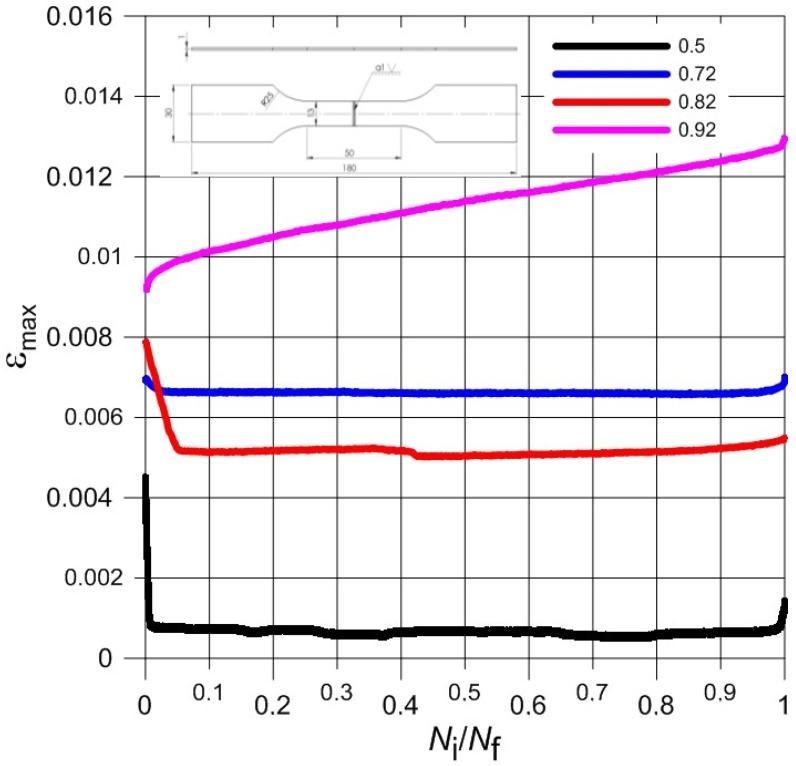
The course of maximum total strain during the fatigue load process of Type 2 samples.

**Figure 7:**
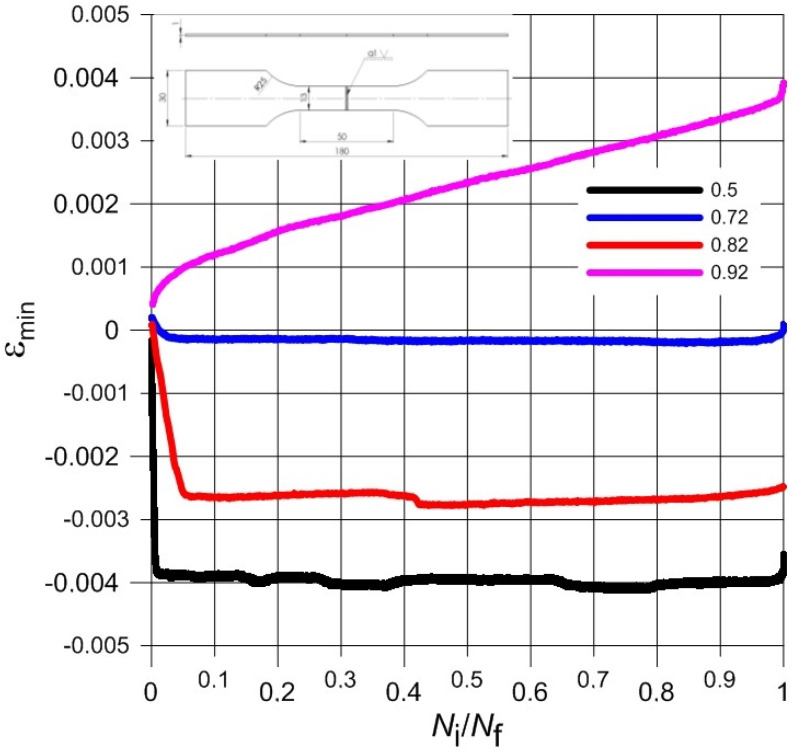
The course of minimum total strain during the fatigue load process of Type 2 samples.

**Figure 8:**
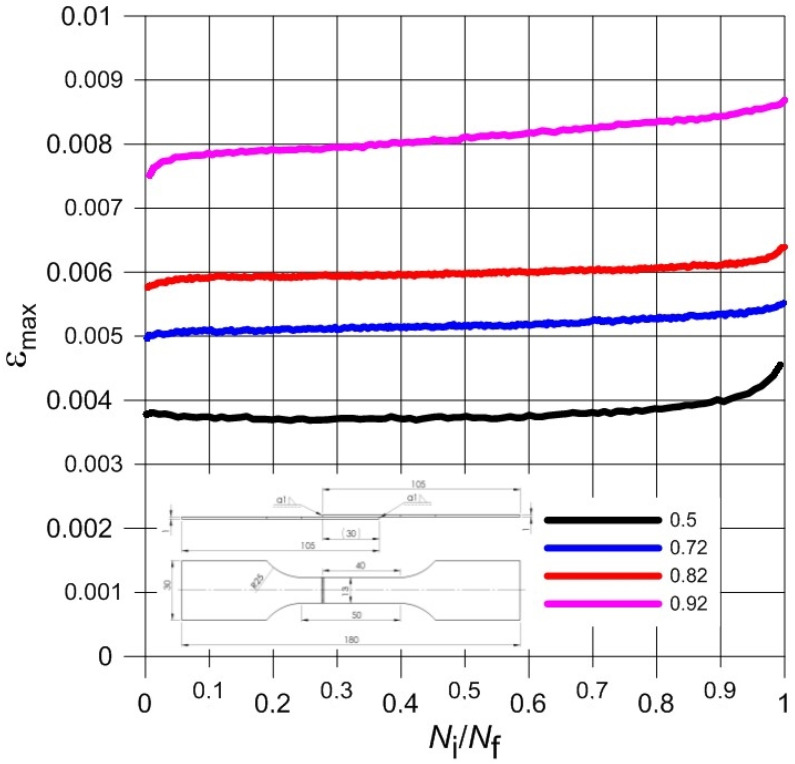
The course of maximum total strain during the fatigue load process of Type 3 samples.

**Figure 9:**
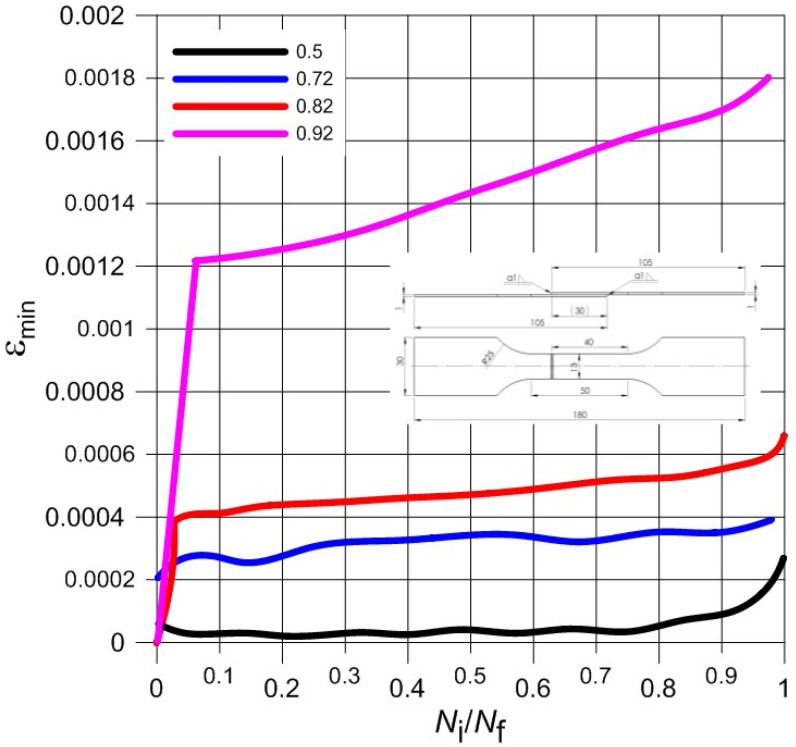
The course of minimum total strain during the fatigue load process of Type 3 samples.

Normalized waveforms of minimum and maximum values of total strain recorded during cyclical loading of samples with respect to the number of cycles were presented in Figure 6, Figure 7, Figure 8 and Figure 9 for both weld types. Generally, the maximum and minimum strain of samples follows a similar course through the testing. However, for type 3 samples, there is an exponential increase in minimum strain in comparison to the maximum strain, which almost remains linear during samples’ fatigue life, especially at higher load levels. In addition, the negative strain values in the unloading cycles can be seen in Table 5. Since the loading was unidirectional and samples were never compressed, this was considered a minor measuring error due to the extensometer. It should be noted that the negative strain values are negligibly small.

Furthermore, the strain values observed from the tests were very low. The difference between the minimum and maximum strain is very small even at highest load of the experiments. Such small strain during fatigue tests seems to coincide with the findings of the monotonic tests; low elongation values in particular. The brittleness of the titanium welded joints is evident and directly influences the fatigue test results negatively. As this research aimed to investigate the low cycle fatigue behaviour primarily, the load levels were chosen accordingly. The number of cycles to fracture was low despite displaying comparable monotonic mechanical properties to base material, especially in the case of Type 3 samples.

**Figure 10:**
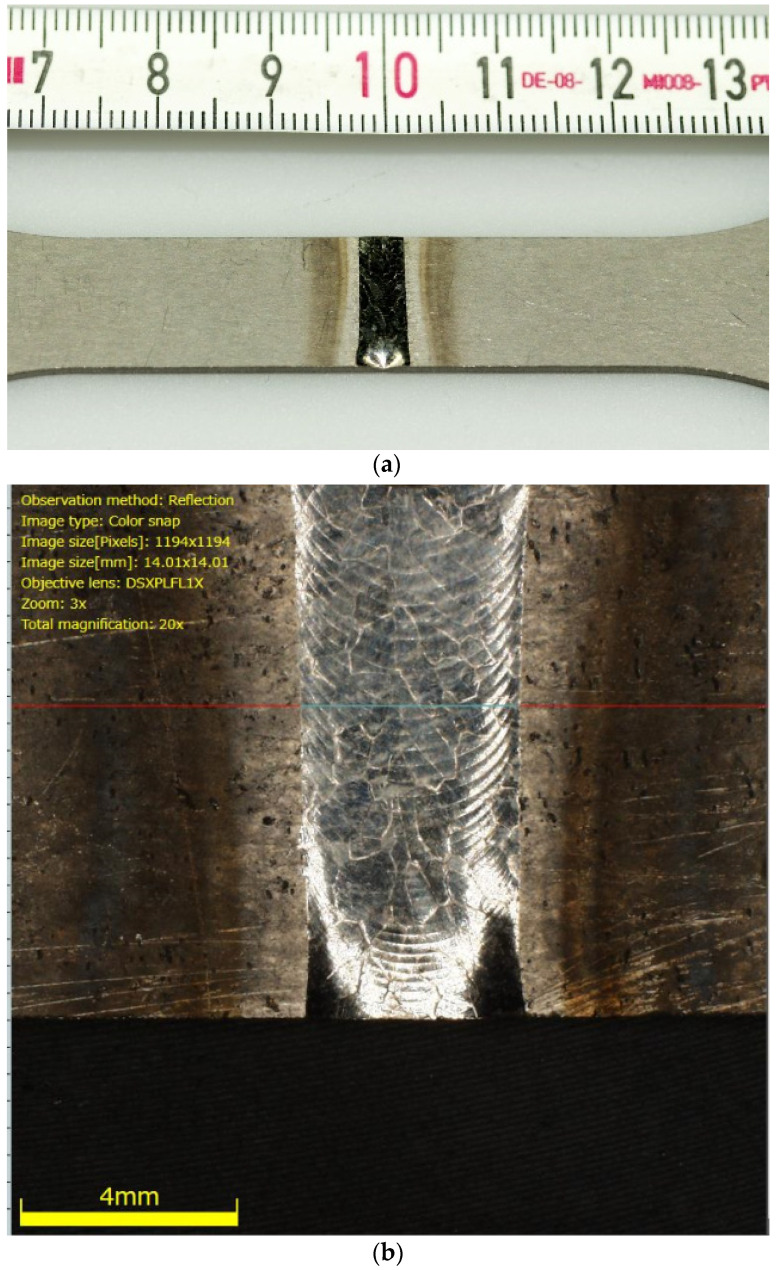
Macroscopic images of Type 2 specimen (**a**) and detailed image of its weld bead; (**b**) before fatigue cracks.

**Figure 11:**
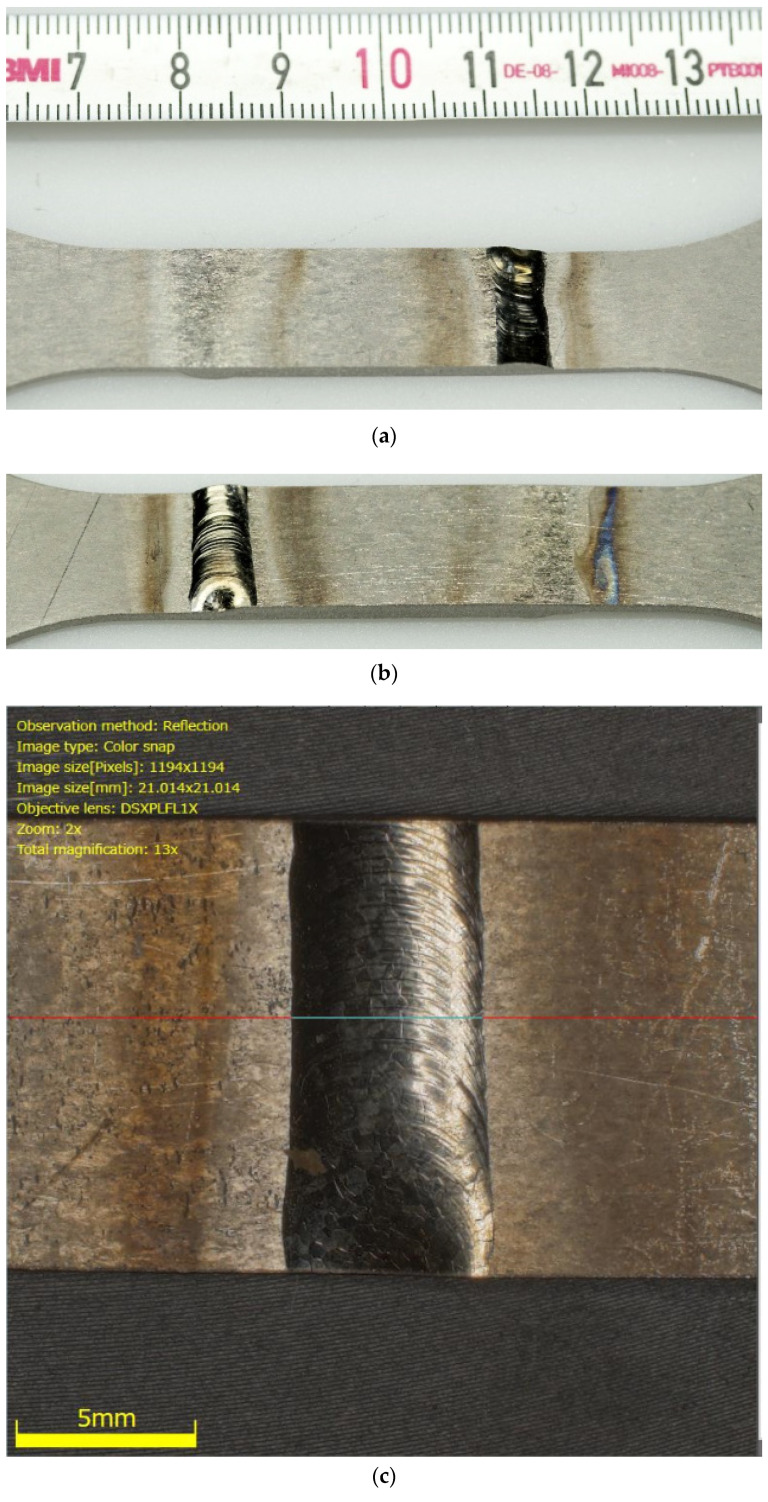
Macroscopic images of Type 3 specimen (**a**,**b**) and detailed image of its weld bead (**c**) before fatigue cracks.

**Figure 12:**
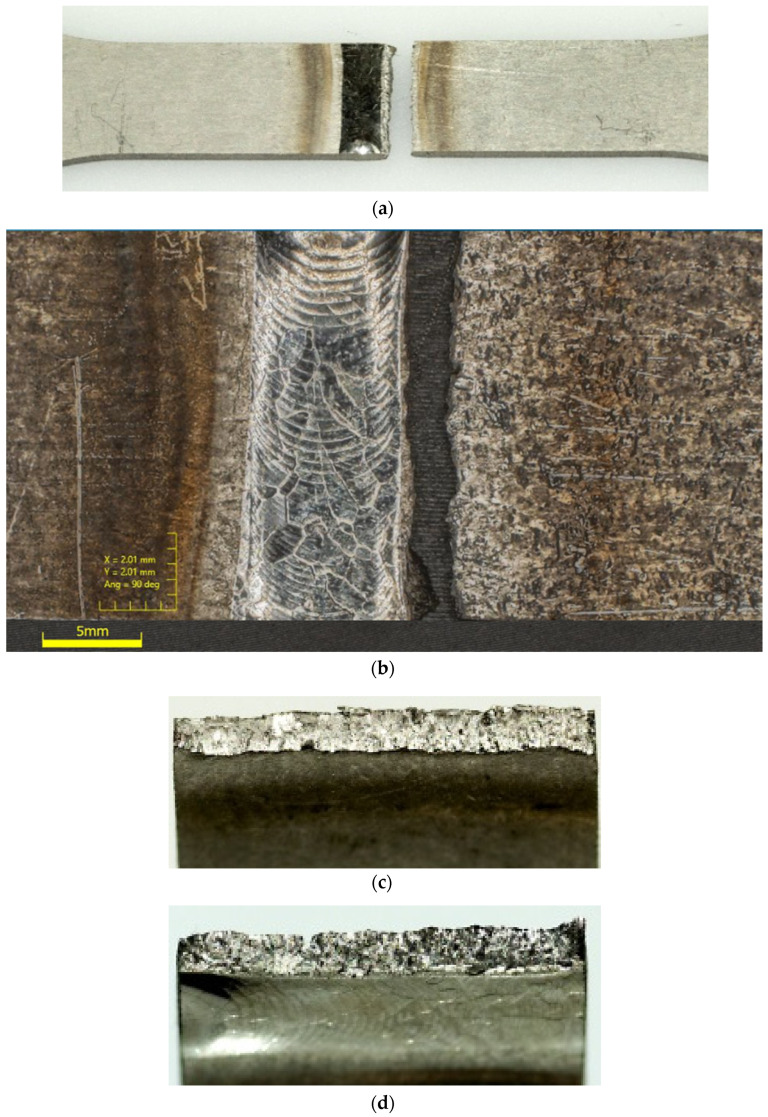
Image of Type 2 specimen (**a**), its weld bead (**b**), after fatigue failure and its fracture surfaces (**c**,**d**).

**Figure 13:**
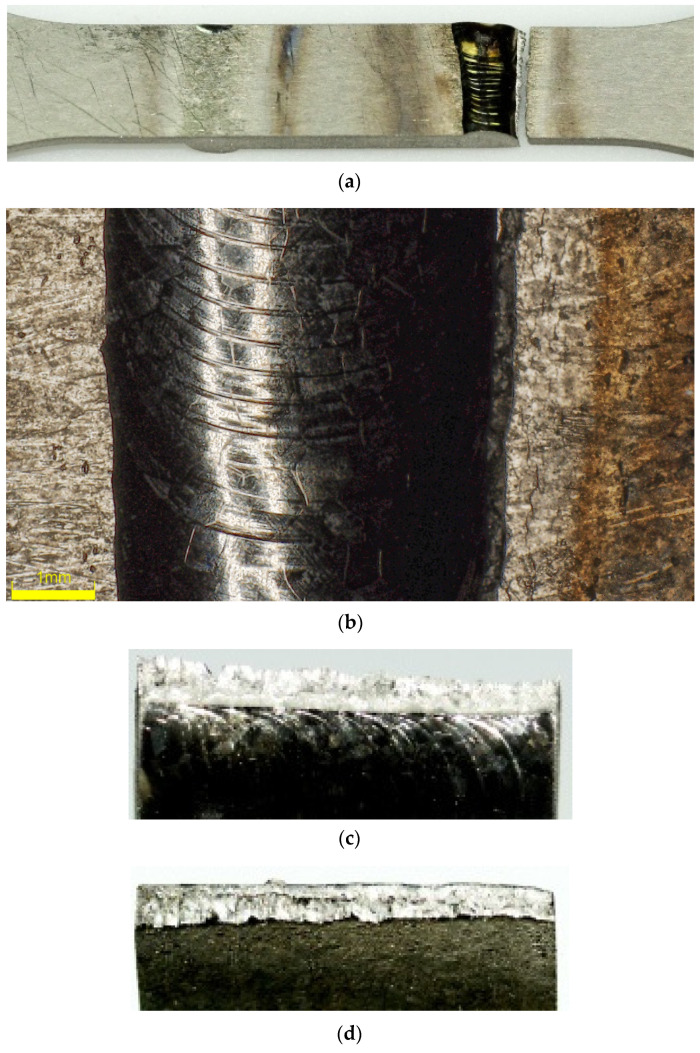
Image of Type 3 specimen (**a**), its weld bead (**b**), after fatigue failure and its fracture surfaces (**c**,**d**).

Despite the precise plasma arc of microplasma arc welding, the adverse effect of welding heat seems to negatively impact the titanium Grade 5 joints. The location of the cracks and fractographic observations further strengthens this conclusion. Figure 10 and Figure 11 present the macroscopic structure of the welds before fatigue tests. From the images, an obvious discolouration around the welds can be observed, which roughly corresponded with the HAZ. Additionally, closer inspection of the surface of the weld bead displayed faint cracks. Since the weld beads were shielded during and after welding, it is presumed that the cracks were formed during rapid cooling of the weld. The fatigue fractures for the analysed types of joints, obtained for a load corresponding to 0.82 $\sigma_{\mathrm{UTS}}$, are presented in Figure 12 and Figure 13. The surfaces of fatigue fractures of welded joints run macroscopically perpendicular to the direction of the load. For the analysed joints, fatigue cracking occurred on the surface of the element in the transition line of the weld face to the welded material, from where the cracking developed. Fatigue fractures have a multi-surface structure with visible focal points. They are responsible for stopping the cracking process or its slowdown as a result of changing load conditions. As the number of load cycles increases, the cracks spread and merge into clusters, creating fatigue breakthrough zones. The progressive development of the crack causes a weakening of the sample cross-section, which will lead to its rapid failure.

**Figure 14:**
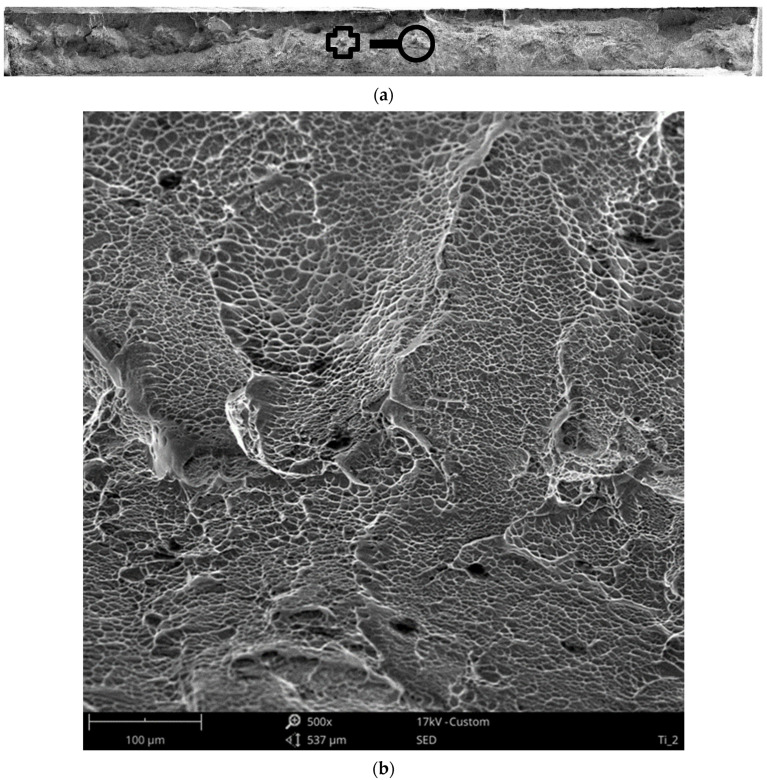

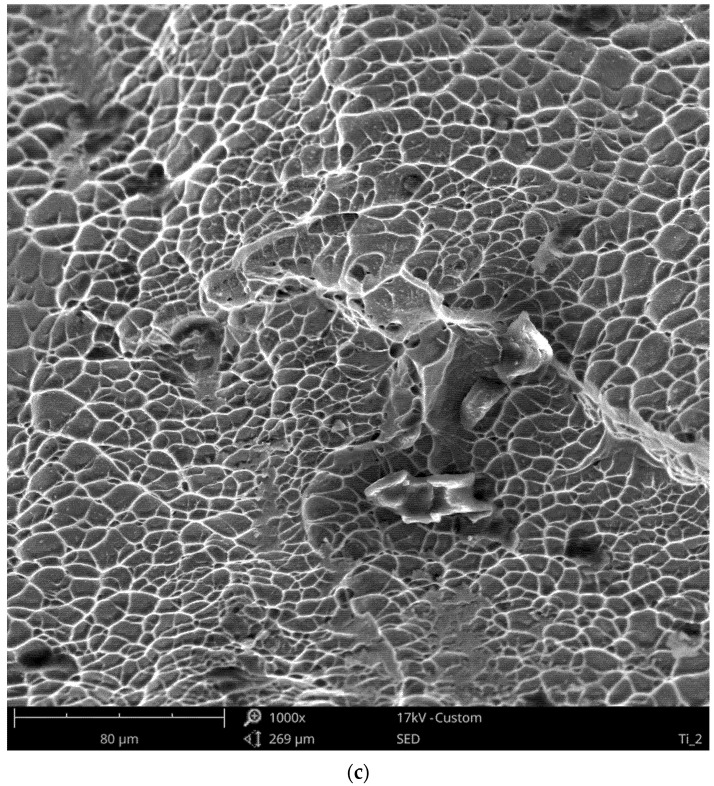

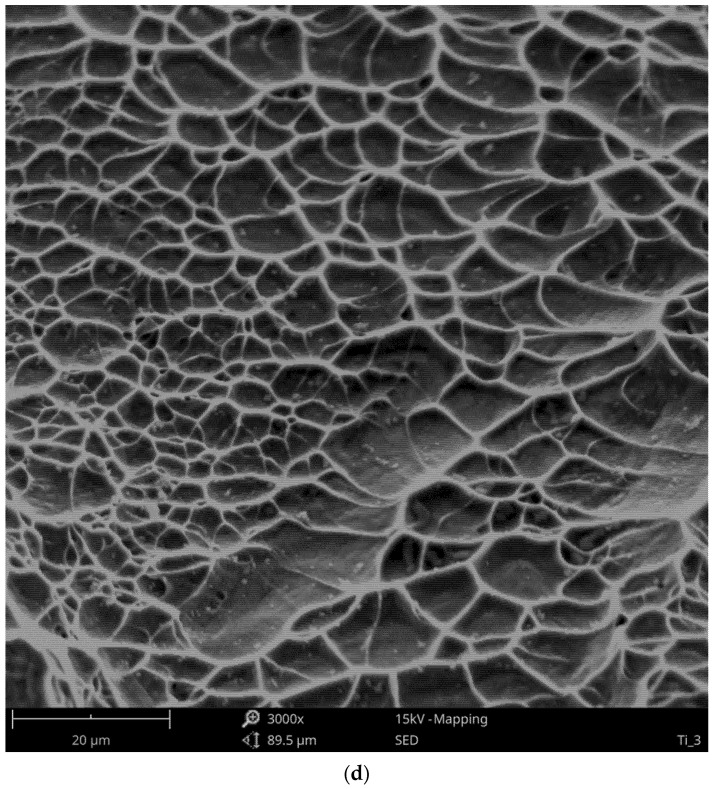
Fracture morphology (**a**) of the titanium weld joint obtained after monotonic loading under different magnifications; ×500 (**b**), ×1000 (**c**), ×3000 (**d**).

**Figure 15:**
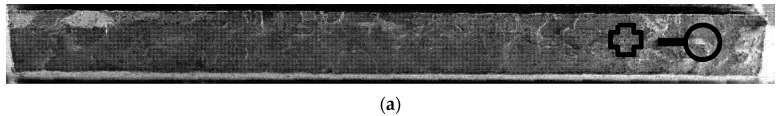

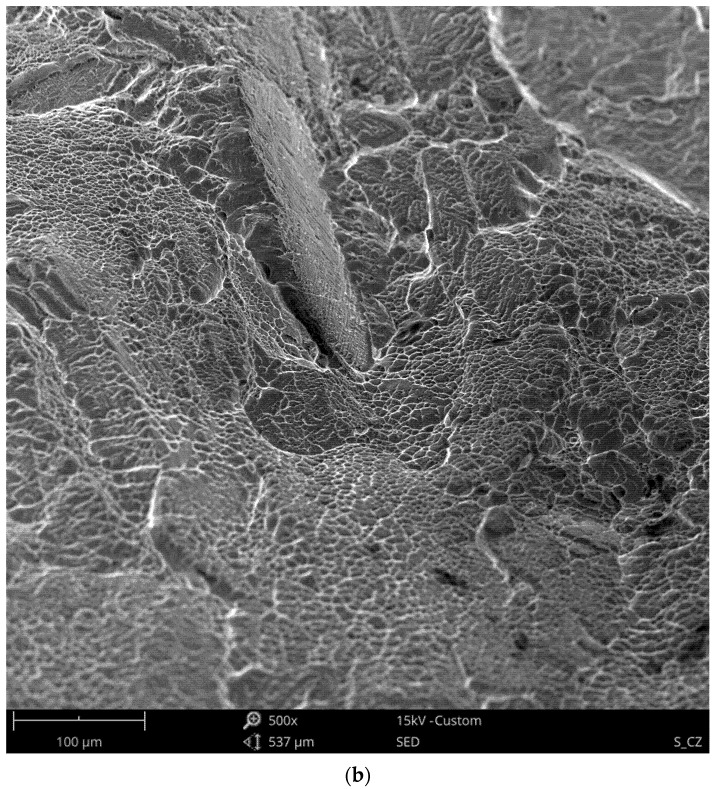

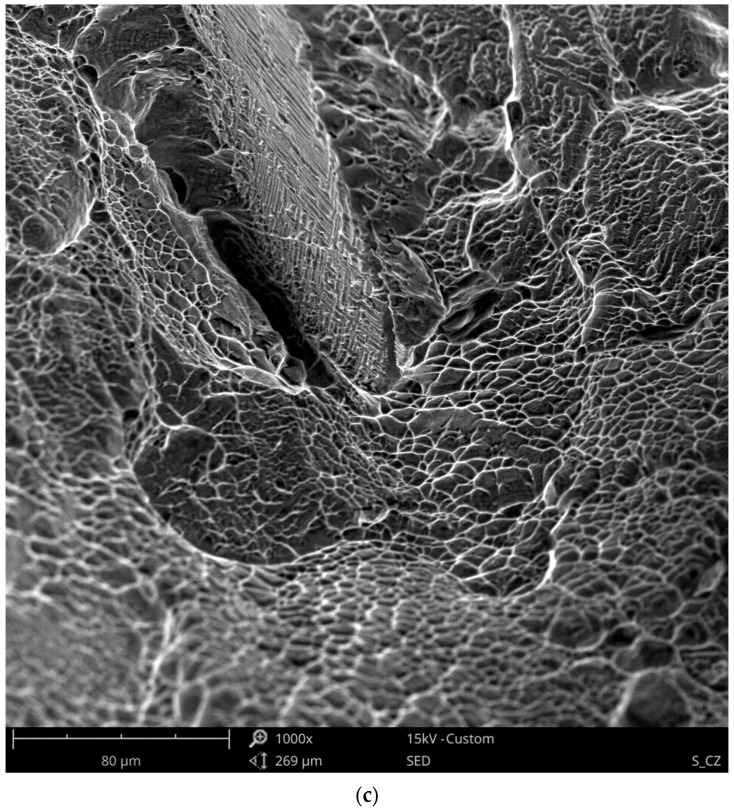

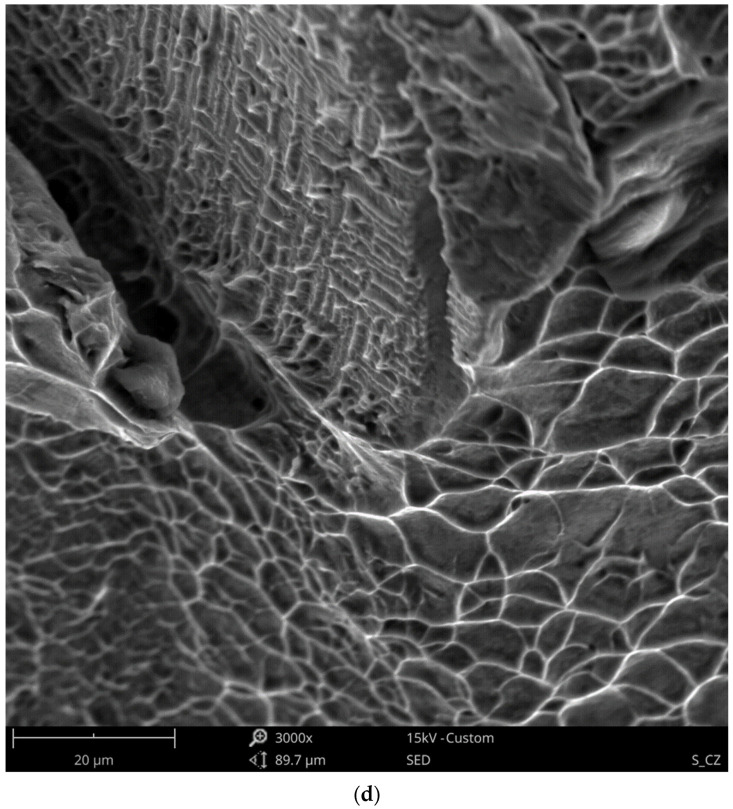
Fracture morphology (**a**) of the titanium weld joint after fatigue tests at 0.82 σ_UTS_ under different magnifications; ×500 (**b**), ×1000 (**c**), ×3000 (**d**).

In order the investigate the mechanism of fatigue cracking in the welded specimens’ butt joint samples (Type 2) under the maximum loading of 0.82 $\sigma_{\mathrm{UTS}}$ was chosen to be observed under the scanning electron microscope (SEM). SEM observations of the samples are presented in Figure 14 and Figure 15. The mechanism of fracture of titanium welded joints was multi-staged. In the first phase, microcracks appeared in the area of structural defects of the joint, which developed from the weld face towards the root. The initial stage of cracking is characterized by a simple morphology of the fracture surface with features of plastic cracking. In the following phase, microcracks started to combine into macro-circulation where the fatigue damage continued to develop at a steady rate. At the fatigue failure, the bands that are typical of this period became visible, which were followed by rapid visible cracking in the heat-affected zone. This formed the visible sharp ridges and dips on the fracture surface, as expected from a brittle fracture. The brittle appearance and location of the fatigue failures further strengthen the implications of previously presented fatigue data. Despite the minimal heat input using the microplasma arc welding, HAZ still plays an integral part in the fatigue behaviour of titanium grade 5 alloy welded joints.

**Figure 16:**
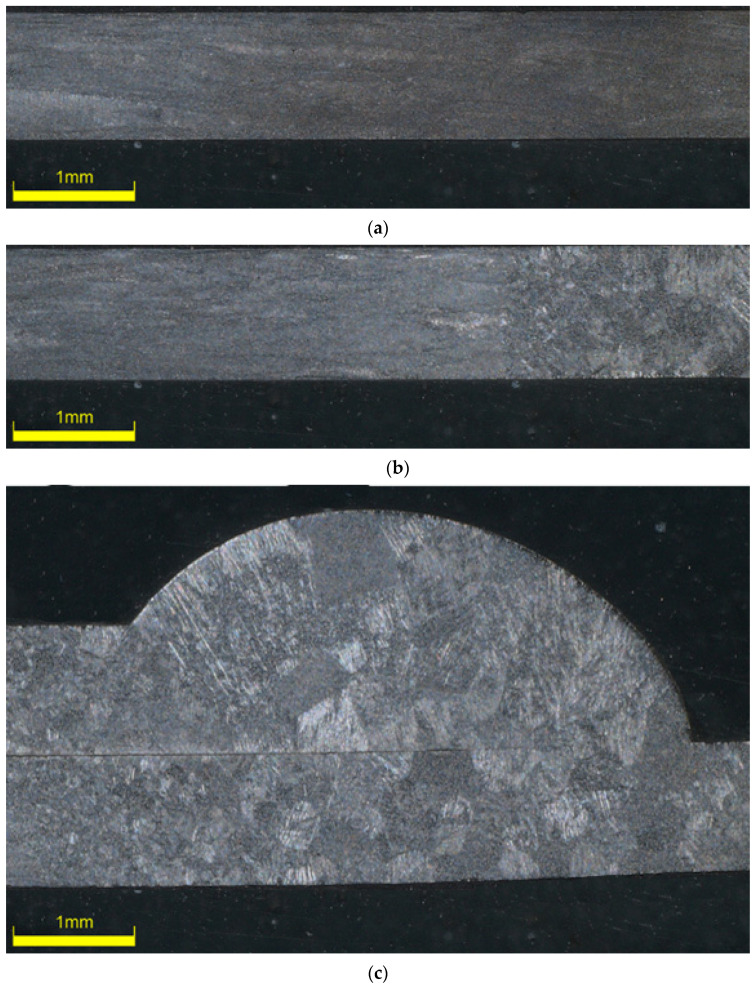
Microstructure of titanium sheet (**a**) and Type 2 (**b**) and Type 3 (**c**) welded samples obtained by microplasma welding at ×30 magnification.

**Figure 17:**
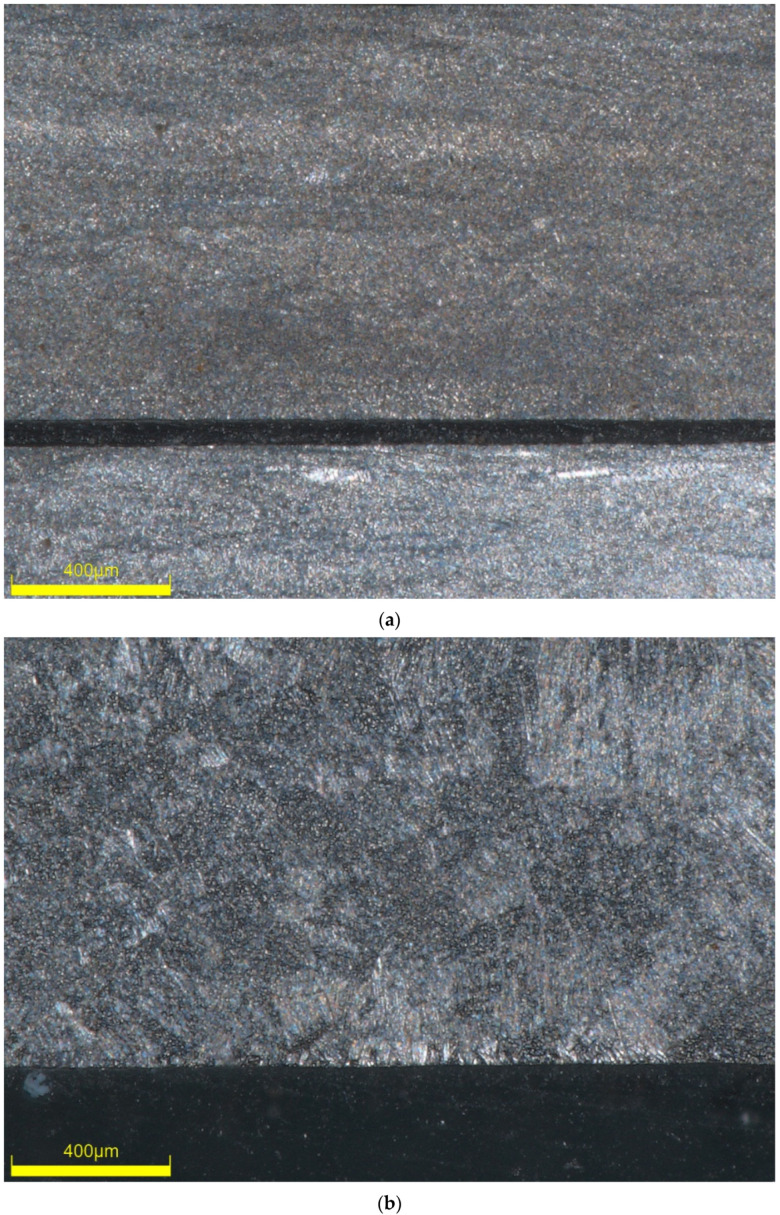

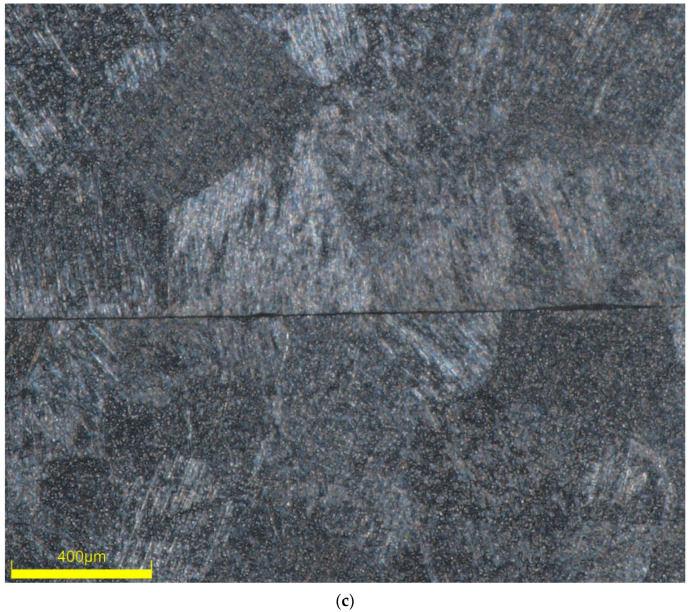
Microstructure of titanium sheet (**a**) and Type 2 (**b**) and Type 3 (**c**) welded samples obtained by microplasma welding at ×145 magnification.

Characteristic microstructures of joints were presented on the cross-section of the tested joints in the form of micrographs at ×30 magnification (Figure 16) and ×145 magnification (Figure 17). Even a quick investigation of welded joints (Figure 16b,c and Figure 17b,c) showed a distinct change in the microstructure of the material. In both welded samples, grain sizes observably increase at the weld beads and gradually decrease along HAZ. This explains the substantial change in the mechanical behaviour of the material both during monotonic and fatigue tests. As expected, increase in the grain size is much more prominent in Type 3 samples (Figure 16c and Figure 17c) due to the increased heat required for a successful fusion. Moreover, microstructural observations show inconsistencies in grains and grain sizes in the HAZ and near-HAZ of welded samples, compared to the relatively homogenous structure of the sample without welds. For Type 2, the effect of HAZ can be observed at the right side of the image (Figure 16b). As for Type 3 samples, showing HAZ alongside unaffected base metal on a single image at this magnification is not possible as it spans the entirety of the image (Figure 16c), which also points to the increased HAZ for Type 3 samples.

Based on all these observations, it was evident that despite the reduced heat input and focused arc beam of the microplasma arc welding, post-welding heat treatments were necessary to improve the fatigue behaviour of titanium Grade 5 welded joints. Low thickness of the samples seem to further amplify these adverse effects. However, it should be noted that the fatigue performance of these samples after post-weld treatment is yet to be investigated.

## 4. Conclusions

Based on the experimental results of monotonic tests, it was possible to successfully weld thin titanium Grade 5 sheets using microplasma arc welding. The strength of these welded joints was comparable to the strength of the base metal. However, the elongation of the material was significantly reduced after welding. This change in material microstructure, which affects the monotonic material behaviour, also seems to persist for the cyclic behaviour of the material. In order to observe the low cycle behaviour of the material, load levels were deliberately chosen close to the ultimate tensile strength of the material. However, even after taking the high level of tensile force into account, fatigue lives of the welded joints were low, especially for Type 3 samples, remaining at an average of 950 cycles under a loading level of 0.5 $\sigma_{\mathrm{UTS}}$, compared to the 28,930 cycles of Type 2 sample under same level of loading. Despite the highly focused heat of microplasma arc, excess welding heat seems to change the material microstructure and greatly reduce the ductility of the material. As Type 3 overlap joint samples was subjected to more heat during the welding of both sides, lowered fatigue lives of the material became a lot more prominent.

In an attempt to thoroughly investigate the present subject, additional research focusing on the fatigue of microplasma arc welded titanium Grade 5 sheets under different loading methods is planned. In addition, methods to improve the monotonic and cyclic properties of the subject material are considered for future studies.

## Figures and Tables

**Table 1:** Analysed chemical composition of test material.

Element.	Net Counts	Weight %	Weight % Error	Atom %	Atom % Error
**Al**	263,482	5.94	±0.03	10.00	±0.05
**Ti**	890,621	93.25	±0.33	88.51	±0.31
**Cr**	2500	0.49	±0.04	0.43	±0.04
**Other**	14,881	0.33	±0.01	1.05	±0.02
**Total**	-	100.00	-	100.00	-

**Table 2:** Welding parameters used in the manufacturing of samples [5].

Parameter	Value	
Current	18	A
Voltage	14.8	V
Welding speed	2	mm/s
Gas flow rate (He)	-	-
Plasma gas	6	L/min
Protective gas	12	L/min
Undercoat gas	20	L/min

**Table 3:** Monotonic material parameters of titanium samples [5].

Sample	E (GPa)	*R*_YP_ (MPa)	*R*_UTS_ (MPa)	*R*_U_ (MPa)	*A* (%)	*Z* (%)
TYPE 1	115	1095	1122	1053	11.5	0.84
TYPE 2	103	1084	1114	1112	3	1.67
TYPE 3	114	1098	1146	1106	1.7	1.51

**Table 4:** Vickers hardness values of titanium welded samples at various locations [5].

Sample	Unaffected Base Metal UBM	Heat Affected Zone HAZ	Weld Centre W
Type 2	358 HV	438 HV	368 HV
Type 3	358 HV	415 HV	344 HV

**Table 5:** List of parameters used during the fatigue test along with corresponding results.

Loading Level	*f* (Hz)	*F*_max_ (N)	*F*_min_ (N)	*σ*_max_ (MPa)	*σ*_min_ (MPa)	*ε*_max_	*ε*_min_	*N*_f_
						in Half of Fatigue Life		
**TYPE 1**								
0.92 *σ*_UTS_	0.5	13,488	0	1032	0	0.009209	0.00113	858
0.82 *σ*_UTS_	0.5	12,024	0	920	0	0.008044	0.00022	2449
0.72 *σ*_UTS_	0.5	10,560	0	808	0	0.007039	0.00019	4983
0.5 *σ*_UTS_	1	7332	0	561	0	0.004893	0.00014	35,529
**TYPE 2**								
0.92 *σ*_UTS_	0.5	13,440	0	1025	0	0.011387	0.00233	566
0.82 *σ*_UTS_	0.5	11,980	0	913	0	0.005053	−0.00276	1111
0.72 *σ*_UTS_	0.5	10,520	0	800	0	0.00661	−0.00017	3798
0.5 *σ*_UTS_	1	7300	0	560	0	0.000694	−0.00392	28,930
**TYPE 3**								
0.92 *σ*_UTS_	0.5	13,910	0	1055	0	0.008114	0.001439	142
0.82 *σ*_UTS_	0.5	12,400	0	940	0	0.005985	0.000464	291
0.72 *σ*_UTS_	0.5	10,890	0	825	0	0.005724	0.000735	408
0.5 *σ*_UTS_	0.5	7560	0	575	0	0.003411	−0.00023	950
